# The Moderating Role of Personal Resources Between Demands and Ill-Being of Romanian Healthcare Professionals in the COVID-19 Pandemic

**DOI:** 10.3389/fpubh.2021.736099

**Published:** 2021-12-23

**Authors:** Ica Secosan, Delia Virga, Zorin Petrisor Crainiceanu, Lavinia Melania Bratu, Tiberiu Bratu

**Affiliations:** ^1^Faculty of Medicine, “Victor Babes” University of Medicine and Pharmacy, Timisoara, Romania; ^2^Department of Psychology, West University of Timisoara, Timisoara, Romania

**Keywords:** psychological capital, frontline healthcare workers, COVID-19, mental health complaints, burnout, anxiety, depression

## Abstract

**Background and Objectives:** The illness caused by the new coronavirus (COVID-19) triggered considerable mental consequences for the medical staff. Our aim was to research whether frontline healthcare workers' positive psychological state—PsyCap—impacts the relationship between anxiety/depression and burnout/mental health complaints.

**Material and Methods:** One hundred twenty-six medical professionals working on the frontline at the Intensive Care Unit and Emergency Department in Romania took validated surveys between March and April 2020. All information was collected online after accessing a link that was received in an email message. The inclusion criteria concerned the categories of healthcare professionals who came into direct contact with patients during the COVID-19 global epidemic through the performed medical act, as well as time spent in the medical field of ICU an EM, namely at least 1 year in the department. We excluded from the research other categories of employees and auxiliary staff, as well as healthcare workers with <1-year experience in the medical field. The moderating role of personal resources (PsyCap) between demands (such as anxiety and depression) and ill-being (burnout and mental health complaints) of healthcare professionals were tested *via* hierarchical multiple regressions.

**Results:** We tested the moderating role of PsyCap on the relation between anxiety and ill-being. The results indicated that high anxiety predicts lower emotional exhaustion and a low level of mental health complaints about Romanian healthcare professionals when PsyCap is high. The moderating role of PsyCap on the relation between depression and ill-being was tested in the second hypothesis. The results indicated that high depression predicts lower inefficacy and a low level of mental health complaints about Romanian healthcare professionals when PsyCap is increased.

**Conclusions:** PsyCap is a crucial variable that may decrease the impact of anxiety and depression on psychological outcomes such as emotional exhaustion, inefficacy, and psychological problems among Romanian medical professionals working on the frontline during the COVID-19 global epidemic. Thus, psychological interventions that help medical staff gain personal resources are appropriate in the context of the COVID-19 pandemic.

## Introduction

COVID-19 has and will continue to have significant consequences on burnout for medical professionals. Moreover, the Intensive Care Unit medical professionals and emergency medical staff are frontline workers fighting against the COVID-19 global epidemic. They are the first ones to come into contact with any patients with symptoms of coronavirus infection. Thus, the epidemic of burnout may have worsened since the COVID-19 outbreak.

Burnout is a state of fatigue, both mental and physical, a person's reaction to long-term stress after facing emotional and interpersonal stress factors at the workplace (1). It is already recognized as an occupational hazard, manifesting through emotional depletion, depersonalization, and low personal achievement. Emotional depletion covers the experience of both mental and physical fatigue. It is linked to an individual's stressful situation, which is linked to a decrease in mental and physical resources. Depersonalization occurs when employees create a mental distance between themselves and their work by experiencing the dehumanization of coworkers, duties, or clients (2). Finally, personal achievement refers to feeling professionally inefficient, leading to the loss of productiveness (3). As one study shows, a relevant proportion of frontline healthcare staff, namely doctors and the nursing staff who worked with patients with COVID-19 reported increased levels of emotional depletion and different levels of depersonalization, and reduced personal accomplishment (4).

Based on the Job-Demands-Resources (JD-R) theory, the absence of organizational and personal abilities reduces the means to cope with high job demands, leading to burnout (5). According to the JD-R theory, both contextual factors such as work pressure, heavy workload, and personal conditions such as emotional, physical, and psychological demands, are equally strong predictors of engagement and job satisfaction. Thus, employees who count on many work-related and personal abilities have the means to cope better with work challenges and experience the feeling of well-being to a great extent (6). Moreover, the JD-R model, which focuses on employees' health and performance *via* burnout, can be applied perfectly to the frontline medical staff in the battle with the pandemic. Hence, a positive psychological state such as PsyCap could aid in the already overwhelmed medical system where burnout is commonly met.

### Anxiety, Burnout, and Mental Health

Studies during the COVID-19 pandemic demonstrated that frontline healthcare workers were experiencing higher levels of anxiety and depression. Anxiety refers to an unpleasant sense of fear characterized by uneasiness derived from anticipating danger, agitation, impatience, difficulty relaxing, trouble concentrating, restlessness, irritability, and difficulty falling asleep. While more and more persons suffer from COVID-19 in Romania, medical assets, including personal protective equipment (PPE), intensive care beds, medications, and ventilators, have at times been limited. Moreover, the fear of contamination, insecurity, and mental distress may predispose the medical staff to considerable emotional strain. As prior studies already reported, as medical clinicians were during SARS or Ebola outbreaks, frontline physicians, medical residents, nursing staff, and public health professionals during the COVID-19 global epidemic are very vulnerable to psychological problems (7). Thus, the sudden outbreak of the COVID-19 global epidemic could be viewed as a type of danger that may negatively affect mental health and produce high levels of anxiety, depression, and stress.

In addition, as one study uncovers, higher anxiety and burnout prevalence rates than previously published literature may be attributed to the COVID-19 pandemic (8). During this pandemic, frontline doctors and nurses must spend considerable time interacting with patients diagnosed with novel coronavirus infections. A recent cross-sectional study found that one factor that was strongly linked to anxiety in a multivariable linear regression was mental depletion (9). Anxiety interferes with physicians' functioning under stress and may long-term affect their well-being (10). Furthermore, since the coronavirus outbreak, frontline healthcare workers such as physicians and the nursing staff, must cope with significant physical and psychological fatigue while working in normal conditions (11), research suggesting that fatigue is not only triggered by the workload involving physical movement, but also by mental labor (12), anxiety being positively correlated with exhaustion (13). Many studies revealed the prevalence of anxiety signs or symptoms and mental fatigue among doctors, medical residents and nurses in the frontline of the battle with the novel coronavirus crisis (14).

### Depression, Burnout, and Mental Health

The studies in this area pointed out that high emotional exhaustion is also correlated with greater mood disruption, such as depression (15). Depression refers to a sense of dysphoria, hopelessness, devaluation of life, self-deprecation, lack of interest or involvement, anhedonia, and inertia (16). As one study showed, since the beginning of the COVID-19 pandemic, medical professionals, namely physicians and the nursing staff, were found to show moderate to very severe levels of depression (17). Thus, during the Sars-Cov2 pandemic, the frontline medical staff may display high levels of work-related distress symptoms, such as mental depletion, anxiety, and depression that, in turn, may also expose the clinicians to several psychological problems (18). As healthcare professionals continue to do their duty on the frontline in this pandemic, it is essential to analyze mental and emotional resources in frontline physicians and nurses to decrease the impact of distress in combating the COVID-19 pandemic. However, an increasing number of healthcare professionals manage to transcend these challenges and achieve high professional fulfillment rather than just burnout mitigation (19). Therefore, analyzing the associated factors of the burnout experienced by frontline healthcare workers is essential to alleviate work-related stress symptoms and improve the well-being of frontline healthcare staff.

### Psychological Capital as Moderator

As past studies have already shown, people with high mental strength are able to adapt to changing challenges and demonstrate psychological stability in the face of adversity (20). One definition of PsyCap could be “an individual's positive psychological state of development characterized by: (1) having confidence (self-efficacy) to take on and put in the necessary effort to succeed at challenging tasks; (2) making a positive attribution (optimism) about succeeding now and in the future; (3) persevering toward goals, and when necessary, redirecting paths to goals (hope) in order to succeed; and (4) when beset by problems and adversity, sustaining and bouncing back and even beyond (resilience) to attain success.” (21).

According to JD-R theory, PsyCap, as personal resource, plays a significant moderator role between job demands and the well-being of the employees. The JD-R theory highlights the role of personal resources in dealing with job demands. Thus, frontline healthcare workers who have high PsyCap will better cope with the increased demands from the workplace during the COVID-19 pandemic. In addition, when job resources are high, factors such as performance and level of well-being are improved. As other studies demonstrated, high levels of PsyCap help manage negative behaviors and emotions correlated with stressful work environments or life situations (22). For example, according to COR theory, PsyCap helps cope with adverse events at the workplace by allowing employees to adapt in the face of adverse events at the workplace by allowing employees to adapt in high job demands. Therefore, a high level of PsyCap moderates the impact of different demands on well-being (23).

As one study shows, high levels of self-efficacy correlate with low levels of burnout, depression, and stress in vascular surgery trainees, suggesting a complex relationship between stress, burnout, and self-efficacy (24). Very optimistic medical professionals are better equipped to cope with stress and show lower vulnerability to psychological risks and exhaustion (25). In the medical profession, resilience was described as an ability to adapt, an ability to develop psychological capital, inner growth, or a sort of endurance (26). Several studies have consistently found that high levels of resilience are correlated with low levels of emotional exhaustion (27). Furthermore, a healthcare professional with higher levels of hope will be able to distinguish and follow the way to success (28). The persons with high PsyCap show better resilience in front of stressful events, tend to be more optimistic, and therefore set plans and pathways to improve the situation (29). Moreover, prior literature showed that PsyCap triggers positive feelings which boost performance and job satisfaction (30).

According to this rationale, we can say that persons showing high PsyCap also show lower vulnerability to anxiety and depression, and therefore can show lower levels of emotional exhaustion, cynicism, inefficacy, and mental health complaints. Unfortunately, not very much is known about whether a factor such as PsyCap may alleviate an employee's assessment of and reaction to stress at the workplace. This is because most previous studies situated PsyCap as a mediator or as a predictor factor merely investigated PsyCap as a moderator factor.

Previous studies already uncovered that PsyCap may help individuals cope with burnout (31, 32). Thus, PsyCap may moderate the relationship of anxiety and burnout in frontline medical professionals during the COVID-19 global epidemic. However, high levels of anxiety may increase burnout in medical professionals by deteriorating their PsyCap. Therefore, interventions should be developed to enhance PsyCap, to reduce anxiety and emotional exhaustion in frontline doctors and nurses.

Scholars demonstrated the relationship of PsyCap and burnout (33), meaning that high mental strength is negatively related to mental fatigue. Thus, PsyCap is a crucial variable that has the potential to decrease healthcare workers' exhaustion and improve physical and mental well-being (34). Moreover, as one study showed, PsyCap is a state-like positive resource that can be changed (35), and the adoption of strategies can reinforce the PsyCap level, which, in turn, may increase the workers' satisfaction with their job and their performance at the workplace (36). As previous studies have already shown, PsyCap is a malleable resource and is open to development through interventions (37).

**Figure 1:**
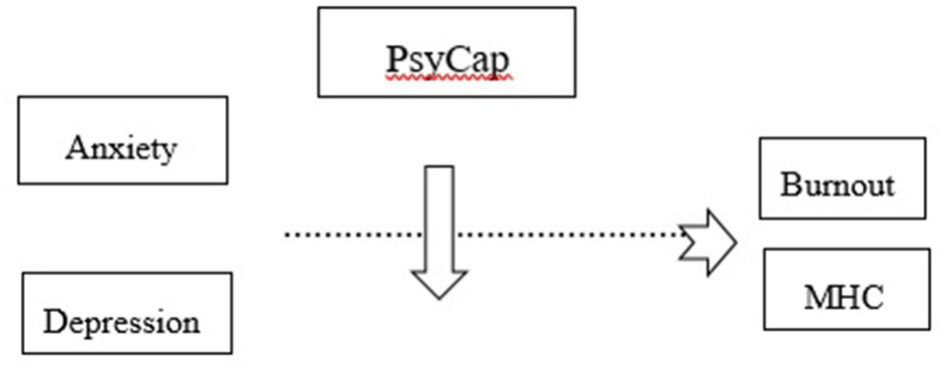
PsyCap moderates the relationship between anxiety/depression and burnout, respectively, mental health complaints.

Hence, our study's contribution strives to understand the PsyCap as a moderator between anxiety, burnout and psychological problems, as well as between depression and burnout, respectively, psychological problems among medical professionals working on the frontline during the COVID-19 global epidemic (Figure 1). Accordingly, we stated the following assumptions:

**Hypothesis 1:** PsyCap moderates the relationship between frontline medical professionals' anxiety during the COVID-19 global epidemic and: (a) emotional exhaustion; (b) cynicism; (c) inefficacy; and (d) mental health complaints.

**Hypothesis 2:** PsyCap moderates the relationship between frontline medical staff's depression during the COVID-19 pandemic and: (a) emotional exhaustion; (b) cynicism; (c) inefficacy; and (d) mental health complaints.

## Participants and Procedure

A cross-sectional study was conducted in the County Emergency Clinical Hospital Pius Brînzeu Timi?oara, Romania, during March and April 2020. The sample contains frontline medical professionals, emergency physicians, ICU physicians, and medical nurses from two Hospital Divisions (Emergency and ICU). The time to complete the survey was 2 months, with four reminders every 2 weeks. In this study, 200 frontline healthcare workers were recruited, and a pool of 126 health professionals (32 nurses and 94 physicians) constituted the study sample (response rate = 63%). Similarities were found between ICU and EM, regarding number of working hours per week, and night shifts per month, no holidays, similar schedule regarding the exposure to COVID-19 patients, similar compensations, guidelines, protocols, and training from the managerial team, access to psychological counseling, similarities in the distribution of the socio-demographic variables, such as gender, marital status, and a number of children.

The research meets the ethical guidelines of the Declaration of Helsinki. The Ethics Committee of the County Emergency Clinical Hospital, No. 170/05.08.2019, approved it as a stage of ongoing research on the exhaustion syndrome and the psychological consequences on the medical professions. All data were collected with a confidential nature, while all the participants expressed their willingness to voluntarily take part in the study and their informed consent in writing. Participants did not receive any incentives, and participation was voluntary.

The participants reported the demographic data, as follows: gender (35.7% male and 64.3% female), marital status (42.8% single, 52.3% married, and 4.7% divorced), children (55.5% yes, 44.4% no), profession (74.6% physician, 25.3% nurse), staff category-doctors (45.2% trainee, 15% specialist, 16.6% primary, 23% other), and specialty (36.5% ICU and 63.4% EM).

Before the beginning of the study, we organized a focus group with respondents from the Emergency and ICU Departments. Our research targeted two Hospital Divisions, working on the frontline battle against the Covid-19 crisis in Romania, Emergency Department and ICU. We communicated with the medical staff directly *via* their institutional emails. A written consent was sent via email and signed by each participant in the study. The frontline healthcare workers answered the questionnaires during their working hours *via* their institutional emails. Only completed questionnaires can be submitted and considered for the present study.

All information was collected online after accessing a link that was received in an email message. Therefore, there were no missing data or invalid responses in our study sample. The inclusion criteria concerned the categories of healthcare professionals who came into direct contact with patients during the COVID-19 global epidemic through the performed medical act, respectively, primary physicians, experts, trainees, ICU, and emergency medicine medical nurses. Another important inclusion criteria is time spent in the medical field of ICU an EM, namely at least 1 year in the department. We excluded from the research other categories of employees and auxiliary staff, as well as healthcare workers with <1-year experience in the medical field.

The County Emergency Clinical Hospital Pius Brînzeu Timişoara, Romania, started receiving the critical patients infected with the new coronavirus by the end of March 2020, following the Romanian Ministry of Health Order number 533/03.29.2020 regarding the Plan of measures for hospitals' preparation in the face of COVID-19 global epidemic. As a result, by May 2020, there were 19,133 patients with COVID-19 in Romania, 98,403 people isolated, and 2993 people officially quarantined. Furthermore, the increase in demand and changes to supply, increase in donning and doffing PPE, redeployment of staff, the restructuring of hospital premises, more work duties, and the implementation of new guidelines and protocols put a heavy psychological pressure on the Romanian frontline medical professionals.

## Materials and Methods

The dimensions of anxiety and depression were assessed on the Depression, Anxiety, and Stress Scale (DASS-21). The Depression, Anxiety, and Stress Scale (DASS 21) is a reliable and suitable questionnaire to assess symptoms of common mental health problems. The essential function of this scale is to assess the severity of the core symptoms of depression, anxiety, and stress, thus it supports our research hypothesis. The DASS-21 is a self-report questionnaire and assesses the severity of the main symptoms of depression, anxiety, and stress. The scale is consisting of 21 items. In the current study, we used the anxiety and depression scale. Each variable has seven items on each subscale: depression (e.g., “It seems that I couldn't experience any positive feeling whatsoever.”; the Cronbach's alpha for this scale on this sample was α = 0.88), anxiety (e.g., “I was worried about the moments in which I might have a panic attack and look like a fool.”; the Cronbach's alpha for this scale on this sample was α = 0.88), and stress. Respondents were asked to evaluate the items on a Likert scale, from 0 (did not apply to me at all) to 3 (applied to me very much) (38).

Psychological Capital was measured with a 12-item Psychological Capital Questionnaire (39) adapted and validated for the Romanian population (40). Psychological Capital Questionnaire is a valid and reliable instrument to assess self-efficacy, hope, resilience, and optimism with good psychometric properties. This questionnaire includes four dimensions: self-efficacy (six items, α = 0.*8*6), hope (six items, α = 0.79), resilience (six items; α = 0.81), and optimism (six items, α = 0.88). The items (e.g., “I'm always optimistic about my future.”; “Failure just makes me try harder.”; “I energetically pursue my goals.”; “I enjoy dealing with new and unusual situations.”) are rated on a 6-point Likert scale, ranging from “strongly disagree” (1 point) to “strongly agree” (6 points). Higher scores indicate higher levels of PsyCap. Cronbach's alpha value of the PsyCap scale on this sample was α = 0.89.

The burnout dimensions were assessed based on the Maslach Burnout Inventory MBI-GS. This tool is a reliable and suitable questionnaire to evaluate the three elements of the burnout syndrome: emotional exhaustion (EE), depersonalization (DP), and inefficacy (IN). Each element consists of 5 items: exhaustion (e.g., “I feel emotionally depleted from my work.”; the Cronbach's alpha for this scale was α = 0.87); cynicism (e.g., “I have become more cynical whether my work has any contribution.”; the Cronbach's alpha for this scale was α = 0.80); and inefficacy (e.g., “I do not deal effectively with people's problems.”; the Cronbach's alpha for this scale was α = 0.78). The items are evaluated on a seven-point scale from 0- “never” to 6- “always” (3).

Psychological problems were screened with the MHI-5 test. This scale consists of five items (e.g., “During the past month, have you felt calm and peaceful? How much of the time?”). The item assessment was performed using a 6-point Likert scale, from 1–“never” to 6– “always.” A high score indicated poor mental health (41). Cronbach's alpha value of the mental health complaints scale on this sample was α = 0.85.

## Data Analysis

The moderating role of personal resources (PsyCap) between demands (such as anxiety or depression) and ill-being (burnout and mental health complaints) of healthcare professionals were tested via hierarchical multiple regressions (42). Anxiety or depression had acted as predictors and burnout and mental health complaints were outcomes. We tested the hypotheses with the help of hierarchical multiple regressions with PsyCap as a moderator. Then, we converted the predictors (anxiety and depression) and the moderator (PsyCap) in *z*-scores and calculated the interaction between predictor × moderator. First, we entered both types of variables in Step 1. After that, Step 2 introduced the predictor, the moderator, and their interaction term. In the first hypothesis (H1), we tested the potential moderating role of PsyCap, as a personal resource, on the relation of anxiety with ill-being. The moderating role of PsyCap on the relation of depression with ill-being was tested in the second hypothesis (H2). We have used the Statistical Package for Social Science (SPSS) v. 21.00 program (IBM Corp., Armonk, N.Y., USA) to test our hypothesis.

## Results

Means, standard deviations, and correlations for the study's variables are shown in Table 1. The data show a positive correlation between anxiety and emotional exhaustion (*r* = 0.70, *p* < 0.001), cynicism (*r* = 0.74, *p* < 0.001), inefficacy (*r* = 0.68, *p* < 0.001), and mental health complaints (*r* = 0.75, *p* < 0.001). We found positive correlation between depression and emotional exhaustion (*r* = 0.71, *p* < 0.001), cynicism (*r* = 0.7, *p* < 0.001), inefficacy (*r* = 0.73, *p* < 0.001), and mental health complaints (*r* = 0.74, *p* < 0.001).

**Table 1:** Means (M), standard deviations (SD), and correlations between the variables (*N* = 126).

	**Variables**	***M***	***SD***	**(1)**	**(2)**	**(3)**	**(4)**	**(5)**	**(6)**	**(7)**	**(8)**	**(9)**
(1)	Anxiety	3.06	3.70	–								
(2)	Depression	4.00	4.16	0.86^**^	–							
(3)	Emotional exhaustion	7.41	6.64	0.70^**^	0.71^**^	–						
(4)	Cynicism	4.60	6.30	0.74^**^	0.77^**^	0.69^**^	–					
(5)	Inefficacy	4.40	6.82	0.68^**^	0.73^**^	0.71^**^	0.77^**^	–				
(6)	Mental health complaints	12.63	5.560	0.75^**^	0.74^**^	0.77^**^	0.64^**^	0.58^**^	–			
(7)	PsyCap	4.54	0.83	−0.53^**^	−0.58^**^	−0.57^**^	−0.53^**^	−0.55^**^	0.61^**^	–		
(8)	Age	34.86	9.68	−0.17^*^	−0.20^*^	−0.22^**^	−0.20^*^	0.27^*^	−0.19^*^	−0.27^*^	–	
(9)	Gender	–	–	0.22^*^	0.25^*^	0.27^**^	0.18^*^	0.12	0.33^*^	0.41^**^	−0.24^*^	–

* *p < 0.05*;

** *p < 0.01*.

After mean-centering all the independent variables, we tested eight separate regression models. The results of these moderator analyses are shown in Tables 2, 3. In Table 2, in Step 1, anxiety positively predicted emotional exhaustion (*β* = 0.69, *p* < 0.001), cynicism (*β* = 0.64, *p* < 0.001), inefficacy (*β* = 0.53, *p* < 0.001), and mental health complaints (*β* = 0.58, *p* < 0.001). Also, PsyCap predicted negatively emotional exhaustion (*β* = −0.20, *p* < 0.001), cynicism (*β* = −0.19, *p* < 0.001), inefficacy (*β* = −0.27, *p* < 0.001), and mental health complaints (*β* = −0.30, *p* < 0.001).

**Table 2:** Hierarchical multiple regression: The alleviating effect of PsyCap in the relationship between anxiety and outcomes (burnout and mental health complaints).

**Steps**	**Predictors**	**Emotional exhaustion**		**Cynicism**		**Inefficacy**		**Mental health complaints**	
1	Anxiety	0.69^**^	0.74^**^	0.64^**^	0.65^**^	0.53^**^	0.48^**^	0.58^**^	0.67^**^
	PsyCap	−0.20^**^	−0.22^**^	−0.19^*^	−0.19^*^	−0.27^**^	−0.25^**^	−0.30^**^	−0.33^**^
2	Anxiety × PsyCap		−0.12^*^		0.02		0.05		0.19^*^
	Total *R*^2^	0.67	0.68	0.58	0.58	0.51	0.52	0.62	0.65
	Δ *R*^2^		0.01		0.00		0.01		0.03
	Final *F*	128.61^**^	3.74^*^	85.32^*^	0.18	65.40^**^	2.44	101.29^**^	9.51^*^

* *p < 0.01*;

** *p < 0.001; N = 126*.

**Table 3:** Hierarchical multiple regression: The alleviating effect of PsyCap in the relationship between depression and outcomes (burnout and mental health complaints).

**Steps**	**Predictors**	**Emotional exhaustion**		**Cynicism**		**Inefficacy**		**Mental health complaints**	
1	Depression	0.72^**^	0.73^**^	0.70^**^	0.69^**^	0.61^**^	0.56^**^	0.73^**^	0.78^**^
	PsyCap	−0.15^*^	−0.15^*^	−0.12	−0.12	−0.19^*^	−0.18^*^	−0.18^**^	−0.20^**^
2	Depression × PsyCap		0.007		−0.01		−0.14^*^		0.14^*^
	Total *R*^2^	0.68	0.68	0.61	0.61	0.56	0.57	0.73	0.75
	Δ *R*^2^		0.00		0.00		0.01		0.02
	Final F	131.96^**^	0.01	96.71^*^	0.06	78.16^**^	4.66^*^	175.97^**^	8.08^*^

* *p < 0.01*;

** *p < 0.001; N = 126*.

**Figure 2:**
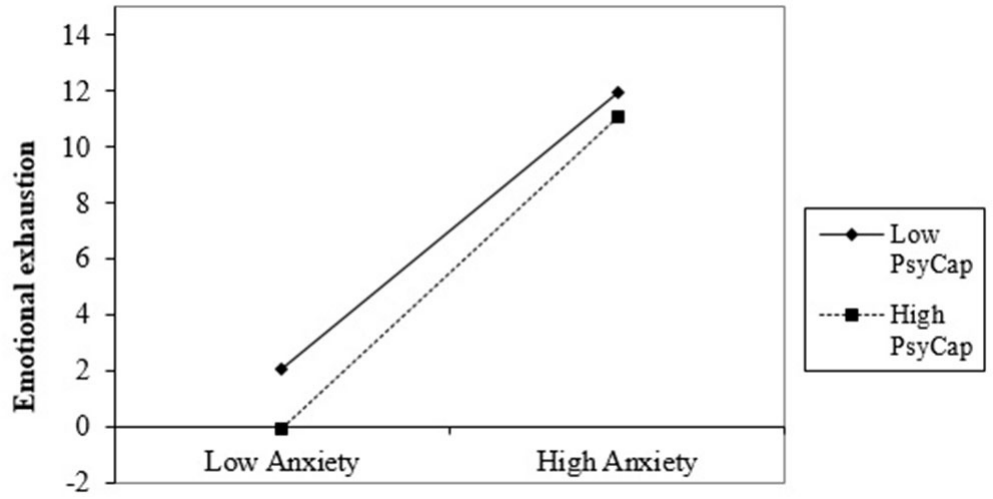
Interaction effect of anxiety and PsyCap in predicting emotional exhaustion.

In Step 2, the interaction between PsyCap and anxiety is not significant for cynicism (*β* = −0.02, *p* > 0.05) and inefficacy (*β* = −0.11, *p* > 0.05), but is significant marginal for emotional exhaustion (*β* = −0.12, *p* < 0.05; *F*_(1,122)_ = 3.74, *p* = 0.05; Δ*R*^2^ = 0.01; 95% CI [0.00, 1.27]) and mental health complaints (*β* = −0.19, *p* < 0.01; *F*_(1,122)_ = 9.51, *p* < 0.01; Δ*R*^2^ =0.03; 95% CI [0.31, 1.45]). As a strategy, for each significant relation, we generated the considerable interaction at ±1 SD from the PsyCap mean (43) and simply analyzed the slopes to determine what kind of interactions occurred (see Figure 2, ).

The simple slope chart in Figure 2 indicated that high anxiety significantly predicts lower emotional exhaustion about Romanian healthcare professionals when PsyCap is high (*b* = 5.58, *t* = 50.53, *p* < 0.001). Also, when healthcare professionals with a low PsyCap face severe anxiety, they experience severe emotional exhaustion (*b* = 4.96, *t* = 11.90, *p* < 0.001).

**Figure 3:**
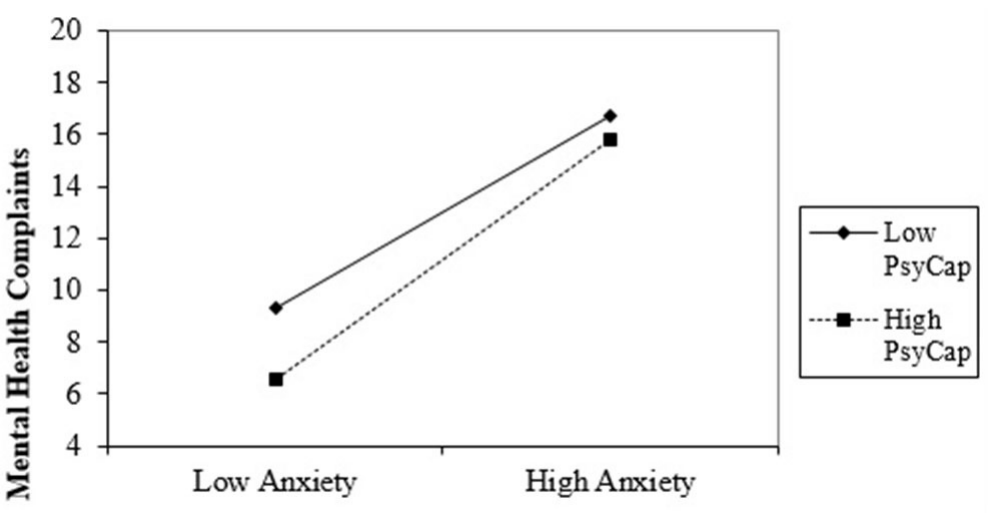
Interaction effect of anxiety and PsyCap in predicting mental health complaints.

The simple slope chart in Figure 3 indicated that high anxiety significantly predicts higher mental health complaints about Romanian healthcare professionals when PsyCap is low (*b* = 3.72, *t* = 8.18, *p* < 0.001). Also, if healthcare professionals with high PsyCap face severe anxiety, they experience fewer psychological problems (*b* = 4.60, *t* = 4.99, *p* < 0.001). Therefore, data also partially supported Hypothesis 1.

As evident in Table 3, in Step 1, depression positively predicted emotional exhaustion (*β* = 0.72, *p* < 0.001), cynicism (*β* = 0.70, *p* < 0.001), inefficacy (*β* = 0.61, *p* < 0.001), and mental health complaints (*β* = 0.73, *p* < 0.001). Also, PsyCap negatively predicted emotional exhaustion (*β* = −0.15, *p* < 0.001), inefficacy (*β* = −0.19, *p* < 0.001), and mental health complaints (*β* = −0.18, *p* < 0.001), but not cynicism (*β* = −0.01, ns.).

In Step 2, there is not significant interaction between PsyCap and depression for emotional exhaustion (*β* = 0.007, *p* > 0.05) and for cynicism (*β* = −0.01, *p* > 0.05), but is significant for inefficacy (*β* = −0.14, *p* = 0.03; *F*_(1,122)_ = 4.66, *p* < 0.01; Δ*R*^2^ = 0.01; 95% CI [−1.51, −0.06]) and mental health complaints (*β* = 0.14, *p* < 0.01; *F*_(1,122)_ = 8.08, *p* < 0.01; Δ*R*^2^ = 0.02; 95% CI [0.19, 1.09]). Also, for each significant relation, we generated the considerable interaction at ±1 SD from the PsyCap mean and simply analyzed the slopes to determine the what kind of interactions occurred.

**Figure 4:**
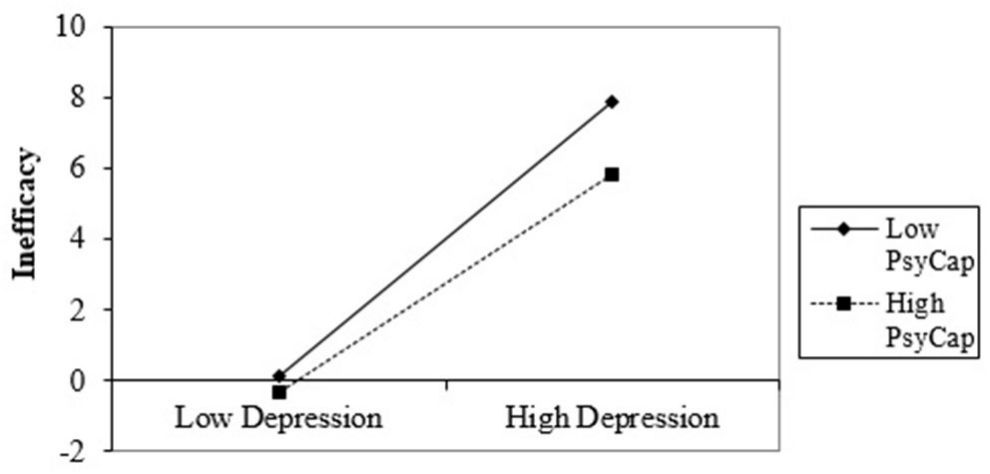
Interaction effect of depression and PsyCap in predicting inefficacy.

The slope chart in Figure 4 indicated that high depression significantly predicts lower inefficacy about Romanian healthcare professionals when PsyCap is increased (*b* = 3.08, *t* = 28.16, *p* < 0.001). Also, if healthcare professionals with low PsyCap face severe depression, they show considerable inefficacy (*b* = 3.87, *t* = 8.65, *p* < 0.001).

**Figure 5:**
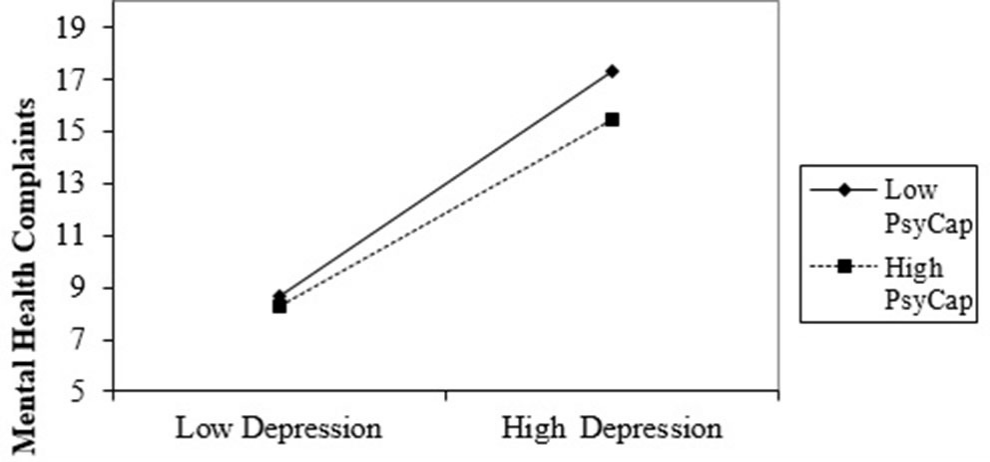
Interaction effect of depression and PsyCap in predicting mental health complaints.

In Figure 5, the simple slope chart indicated that high depression significantly predicts higher mental health complaints about Romanian healthcare professionals when PsyCap is low (*b* = 4.36, *t* = 9.49, *p* < 0.001). Also, if healthcare professionals with high PsyCap face severe depression, they have fewer psychological problems (*b* = 3.57, *t* = 32.58, *p* < 0.001). Thus, Hypothesis 2 was also partially supported by the data.

## Conclusion and Discussion

Since the outbreak of the COVID-19 pandemic, frontline healthcare professionals have experienced unprecedented levels of work-related stress symptoms alongside other psychological implications. Therefore, it is of most importance to determine psychological actions focused on the mental health consequences of the COVID-19 global pandemic, as well as on the moderators of the correlation between anxiety and depression and burnout, respectively, mental health complaints. This study's contribution is to shift the attention on capital, namely PsyCap, rather than shortfalls or challenges when conducting the research on the frontline healthcare workers during the novel coronavirus pandemic. Our study shows PsyCap as a primary variable in decreasing the effect of anxiety and depression on burnout and mental health complaints. Our findings are congruent with prior studies that demonstrated PsyCap's alleviating role in job-related circumstances (44). The purpose of our research was to determine whether the positive mental state of frontline healthcare workers—PsyCap—influences the correlation between anxiety/depression and burnout/mental health complaints. The results showed a partial concordance with our assumptions.

First, we noticed that a high level of anxiety significantly predicts lower emotional exhaustion when the level of PsyCap is high. Furthermore, when healthcare workers with a low PsyCap face severe anxiety, they experience severe emotional exhaustion. Previous studies have already shown that workers with high levels of PsyCap tend to have more confidence in mobilizing motivation and cognitive resources (45). Furthermore, faced with *uncertain situations*, healthcare workers with high PsyCap are more resilient, able to recover from problems, and follow the way to success (46). However, if frontline health staff is less optimistic, motivated, and resilient at work, they are more prone to experience emotional exhaustion when confronted with anxiety. Our results align with the JD-R theory (47), which suggests that inner abilities, namely hope, optimism, resilience, and self-efficacy, may alleviate the ways employees experience burnout.

Second, we observed that a high level of anxiety also predicts higher mental health complaints when PsyCap is low. Moreover, when frontline health professionals with high PsyCap face severe anxiety, they show fewer psychological problems. These results are relevant in the context of JD-R theory; a high level of PsyCap helps cope with anxiety and diminish the impact on mental health complaints. This theory indicates that individuals strive to accumulate and preserve resources (48), and therefore, employees with high levels of PsyCap have confidence in their ability to cope with job challenges. Consequently, frontline healthcare workers who are resilient when experiencing stressful job demands show low mental health complaints.

Third, the results revealed that a high level of depression significantly predicts lower inefficacy when PsyCap is high. Furthermore, when frontline healthcare workers with a low PsyCap face severe depression, they become highly inefficient. This finding supports earlier research reporting that PsyCap is positively correlated with performance (49). Thus, PsyCap could play a decisive role in efficacy at work. Romanian frontline doctors and nurses with high levels of personal resources can handle challenges at the workplace, such as depression in the context of the COVID-19 pandemic, and, therefore, have control over their work environment and display efficacy.

Fourth, we found that a high level of depression significantly predicts higher mental health complaints when PsyCap is low. When frontline health staff with high PsyCap faces severe depression, they show fewer psychological problems. According to a meta-analysis already conducted, PsyCap is correlated with health as an element of well-being (50). These findings support previous studies that operationalized PsyCap as an alleviating variable correlated with other variables (51). In the context of JD-R theory, personal resources, such as PsyCap, have a vital role in connecting depression and psychological complaints.

The outbreak of COVID-19 caused considerable psychological implications for frontline healthcare workers. The medical population working on the frontline in the battle with the novel coronavirus displays a high prevalence of stress symptoms, anxiety, depression, which may predispose the clinicians to various other mental health problems. Our research revealed PsyCap as a significant variable in alleviating the impact of anxiety and depression on emotional exhaustion, inefficacy, and mental health complaints. These results are in line with previous empirical studies and conceptual models that supported PsyCap's alleviating role in supporting positive personal and organizational consequences (52).

### Limitations

Our aim was to study the moderating role of PsyCap in the relationship between demands and ill-being; further research may contribute to better understanding ways in which the causality relationship regarding anxiety, depression, mental health complaints, burnout, and PsyCap may occur. There are several limits in our study. First, as it was designed as a cross-sectional study, it could not establish the relationship between variables. A further contribution could be obtained through future longitudinal research that could lead to better knowledge of causality between the variables. Second, the study variables were measured by a self-reported questionnaire, which could have had an impact on the results as a consequence of the common bias-variance method. Third, our sample size was too small, therefore, the results might not actually depict the entire situation in Romania. Moreover, further research could expand this topic and investigate the role of PsyCap on other frontline healthcare worker's personal and organizational implications. Further research with a larger sample of participants, such as a nation-wide study should be performed, in order for the results to be completely representative of the situation in Romania and obtain a complete picture of all the short- and long-term health implications on frontline healthcare workers, since the outbreak of COVID-19 crisis. Moreover, further research considering an analysis with structural equations may bring more clarity among the studied variables, if we want to test the mediator role of the PsyCap between demands and ill-being.

### Practical Implications

Our results suggest that the significant psychological implications of the COVID-19 pandemic on the frontline healthcare workers, such as anxiety and depression, may have a reduced impact on other consequences, such as emotional exhaustion, inefficacy, and psychological problems based on medical professionals' personal resources, namely PsyCap. Furthermore, those abilities that seem to matter in the ill-being of the health system's staff are flexible. PsyCap can be changed and developed, hence viewed as a state-like variable (53). As one meta-analysis of controlled interventions highlighted, PsyCap allows changes through sessions, coaching programs, and drills at the workplace. PsyCap is the most suitable personal resource to be developed through such interventions (54).

Hence, psychological interventions that help health staff gain personal resources are appropriate during the COVID-19 global epidemic. Moreover, online sessions focused on increasing PsyCap employees have already proved effective before the pandemic (55). Thus, our research suggests that interventions that model PsyCap are appropriate for frontline healthcare workers during this pandemic. Thereby, interventions focused on increasing the PsyCap of frontline medical staff may prove effective in managing anxiety, depression, and work-related stress since the outbreak of COVID-19 global epidemic.

In conclusion, we consider that our research adds to the recent efforts to discover the role of PsyCap as a moderator in the correlation between anxiety/depression and burnout/psychological problems among health professionals working on the frontline since the outbreak of the COVID-19 global epidemic. Furthermore, these results encourage developing human resource management practices, such as programs to increase PsyCap's performance and health for frontline healthcare workers.

## Data Availability Statement

The original contributions presented in the study are included in the article/supplementary material, further inquiries can be directed to the corresponding author.

## Ethics Statement

The studies involving human participants were reviewed and approved by the Ethics Committee of the County Emergency Clinical Hospital, No. 170/05.08.2019. The patients/participants provided their written informed consent to participate in this study.

## Author Contributions

IS, DV, and LB: conceptualization and resources. IS, ZC, and DV: methodology. DV: software. IS, ZC, DV, and TB: validation. IS, DV, TB, and LB: formal analysis. IS and DV: investigation, writing—original draft preparation, data curation and project administration. IS, DV, and TB: writing—review and editing. IS, ZC, DV, TB, and LB: visualization. TB: supervision. All authors have read and agreed to the published version of the manuscript.

## Conflict of Interest

The authors declare that the research was conducted in the absence of any commercial or financial relationships that could be construed as a potential conflict of interest.

## Publisher's Note

All claims expressed in this article are solely those of the authors and do not necessarily represent those of their affiliated organizations, or those of the publisher, the editors and the reviewers. Any product that may be evaluated in this article, or claim that may be made by its manufacturer, is not guaranteed or endorsed by the publisher.

## Abbreviations

COVID 19: Coronavirus disease
ICU: Intensive Care Unit
PsyCap: Psychological Capital
JD-R: Job-Demands Resources
PPE: Personal protective equipment
SARS: Severe Acute Respiratory Syndrome
The DASS-21: -DepressionAnxiety and Stress Scale
MBI-GS: Maslach Burnout Inventory General Survey
EE: Emotional exhaustion
DP: Depersonalization
IN: Inefficacy
MHI: Mental Health Complaints Inventory.
